# Case Report: A case of *PLA2G6* gene-related early-onset Parkinson's disease and review of literature

**DOI:** 10.3389/fnins.2022.1064566

**Published:** 2022-12-09

**Authors:** Lili Gao, Chunlan Shi, Qing Lin, Yujing Wu, Liqi Hu, Mingwang Wang, Jianhua Guan, Sheng Lin, Yuansheng Liao, Chenghan Wu

**Affiliations:** ^1^Department of Neurology, The Second Affiliated Hospital of Fujian Traditional Chinese Medical University, Fujian, China; ^2^Department of Neurology, Fujian University of Traditional Chinese Medicine, Fujian, China

**Keywords:** EOPD, PLA2G6, PARK14, PD, heterozygous mutation, case report

## Abstract

**Background:**

Early onset Parkinson's disease (EOPD) is a neurodegenerative disease associated with the action ofto genetic factors. A mutated phospholipase A2 type VI gene (*PLA2G6*) is considered to be one of pathogenic genes involved in EOPD development. Although EOPD caused by a mutated *PLA2G6* has been recorded in major databases, not all mutant genotypes have been reported. Here, we report a case of *PLA2G6*-related EOPD caused by a novel compound heterozygous mutation.

**Case presentation:**

The case was an of 26-year-old young male with a 2-year course of disease. The onset of the disease was insidious and developed gradually. The patient presented with unsteady walking, bradykinesia, unresponsiveness, and decreased facial expression. Auxiliary examination showed a compound heterozygous mutation of the *PLA2G6*gene with c.991G > T and c.1427 + 1G > A. Mild atrophy of the cerebrum and cerebellum was detected on brain MRI. The patient was diagnosed with EOPD. We administered treatment with Madopar, which was effective. After a two-year disease course, we observed progression to stage 5 according to the Hoehn-Yahr Scale (without medicine in the off-stage). An MDS-UPDRS III score of 62 was obtained, with characteristics of severe disease and rapid progress. The diagnosis was an EOPD phenotype caused by a combination of mutations at the c.991G > T and c.1427 + 1G > A sites of the *PLA2G6*gene.

**Conclusion:**

After active treatment, the disease was set under control, with no significant progression during the three-month follow-up period. Dyskinesia did not recur after reducing the Madopar dose. The freezing sign was slightly decreased and the wearing-off was delayed to 2 h.

## Introduction

Parkinson's disease (PD) is a multifactorial disease, and its pathogenesis may be related to factors, such as oxidative stress, mitochondrial dysfunction, and abnormal protein expression. However, from 10 to 15% of PD patients have a genetic predisposition (Zhu et al., 2019). Early-onset PD (EOPD) refers to an earlier age onset (50 or younger) of this disease. EOPD is related mainly to more than 20 gene variants such as *ATP13A2, PLA2G6*, and *PRKN* (Zhao et al., 2020). A disease caused by mutation of *PLA2G6* is highly genetically and clinically heterogeneous. The clinical manifestations of *PLA2G6*-related EOPD include mental disorders and/or cognitive decline, non-motor symptoms such as dystonia, and motor symptoms such as resting tremor, muscle rigidity, and bradykinesia (Chan et al., 2008). Although the incidence of EOPD caused by a *PLA2G6* mutation is not high, it has a very high disability and mortality rate, and the average age of onset is 22 years (Oliveira et al., 2021). These disease characteristics bring a heavy burden to patients and families with no curative treatment currently available. Therefore, *PLA2G6-*related EOPD needs more extensive in-depth research. Here, we report a case of EOPD associated with a heterozygous mutation of *PLA2G6*.

## Case description

### Medical history

A 26-year-old male patient was admitted to the Second People's Hospital Affiliated to Fujian University of Traditional Chinese Medicine (Fuzhou, Fujian, China) in May 2022 due to unsteady walking for more than 2 years with progressive aggravation, and hand tremor for more than 1 year. We reviewed his medical history and established that disease symptoms, such as unstable walking, slow movement, and decreased facial expression, gradually developed in early 2020. In April 2020, he attended a local hospital, where he was diagnosed with Parkinson's syndrome. Then, he received treatment with Madopar at 0.125 g tid, which improved the unstable walking and bradykinesia, but not the facial expression. At the end of 2020, the unstable walking and the bradykinesia significantly progressed, and symptoms such as trunk leaning back and tremor of hands (mainly postural maintenance tremor) occurred. The patient increased the amount of Madopar by himself (details unknown). However, involuntary convulsions, outstretched tongue, and other abnormal movement symptoms appeared. These symptoms gradually progressed along with the appearance of freezing symptoms such as inability to move and turn over voluntarily. Thus, the patient gradually increased further the amount of Madopar by himself, up to 1.25 g/day. In April 2022, the patient's off-stage condition progressed to stage 5 with retarded speech, resting tremors in both hands, and the gradual appearance of the “masked face” syndrome of PD. Moreover, he also had by Madopar-induced dyskinesia, freezing symptoms, and wearing off. He was married with two sons and denied family history of genetic diseases and similar diseases. The two sons did not suffer from similar symptoms.

### Physical examination

Blood pressure in lying and standing position was normal. The off-stage nervous system examination showed clear consciousness, unresponsiveness, dysfluent speech, reduced facial expression, normal higher cortical function by rough test, normal bilateral eye movements, grade V of limb strength, increased muscle tone of the right upper and lower limbs (1 score), normal left limb muscle tone, tendon reflexes of the limbs (-), and bilateral pathological signs (-). Besides, the results of the bilateral rotation, the finger-finger test, and the foot tapping were normal. The patient could not cooperate to complete the bilateral finger-nose and heel-knee-shin tests with Romberg's sign (+) and back pull test (+). Support was required during walking. The pace of the case was reduced with lower limbs dragging.

### Laboratory tests

**Figure 1:**
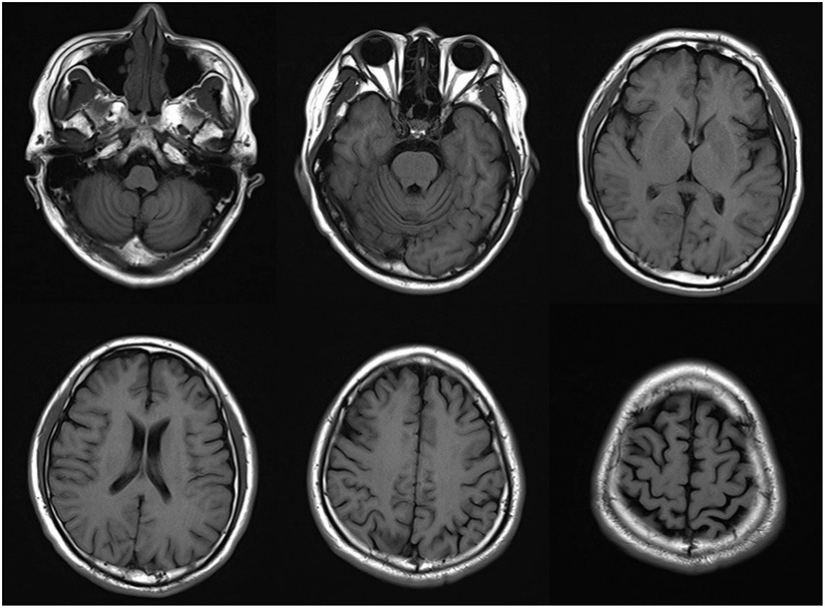
Brain MRI showed atrophy of the cerebrum and cerebellum.

The thyroid function was assessed with the following results: free thyroid hormone level was 36.940 pmol/L and anti-thyroid peroxidase antibody > 1300.0 IU/mL. Blood, urine, stool routine, complete biochemical items, homocysteine, ceruloplasmin, hepatitis, syphilis, HIV, coagulation, and rheumatism indexes were normal. Brain MRI showed atrophy of the cerebrum and cerebellum (Figure 1). EEG indicated moderate abnormality. Electromyography revealed no peripheral neurogenic damage. SEP of the right lower extremity was generally normal. Ophthalmological examination showed no obvious abnormality. The following examination scores were received: AD8, 3; Mini-Mental State Examination (MMSE) 24; Montreal Cognitive Assay (MoCA), 20; HAMD, 49 scores; and HAMA, 23. The Hoehn-Yahr Scale showed stage 5 (without medicine in the off-stage). The following MDS-UPDRS I scores were obtained: MDS-UPDRS I, 8; MDS-UPDRS II, 38; MDS-UPDRS III, 62, and MDS-UPDRS IV, 7. MRI showed atrophy of the cerebrum and cerebellum.

### Genetic test

**Figure 2:**
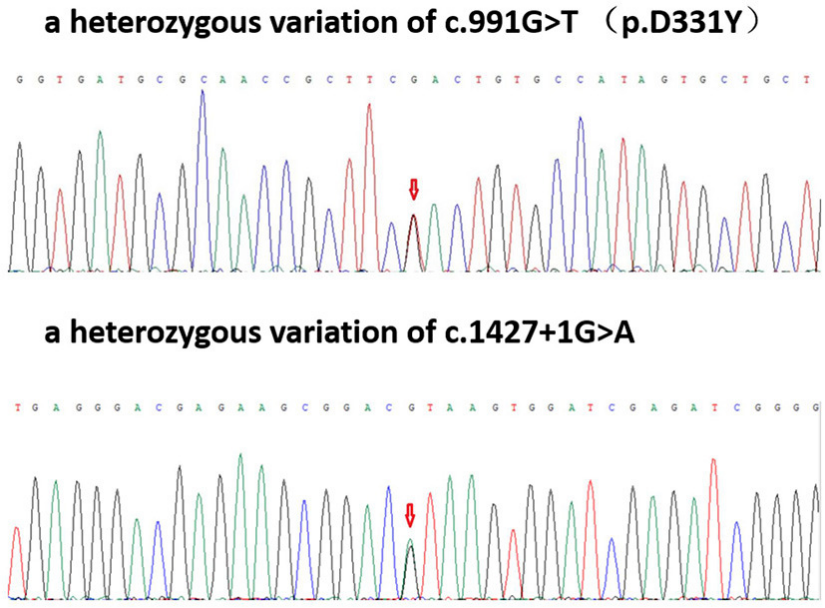
Patient gene map: A heterozygous variation of c.991G > T (p.D331Y); a heterozygous variation of c.1427 + 1G>A.

Two heterozygous mutations were found in the exon and a splice site region of PLA2G6 including c.991G > T (guanine > thymine), which led to p.D331Y (aspartic acid > tyrosine) and c.1427 + 1G > A (guanine > adenine) (Figure 2).

### Diagnosis, treatment, and follow-up period

Based on the patient's symptoms, including bradykinesia, unsteady walking, tremor of hands, and decreased facial expression, the disease was located in the extrapyramidal system. The qualitative diagnosis findings were as follows: factors, such as poisoning, trauma, and infection were denied. According to the characteristics of insidious onset in adolescence and the gradual progression, combined with the genetic test results, the disorder was considered hereditary; the final conclusion was genetic Parkinson's disease type 14 (PARK14). Based on the PD diagnostic criteria of International Parkinson and Movement Disorder Society (MDS) in 2015 (Postuma et al., 2015), the case was diagnosed as EOPD. The following treatment was administered: Madopar 0.25 g tid combined with ropinirole hydrochloride tablets 0.25 g tid. The patient's dyskinesia episodes were significantly less than at the onset, and the motor symptoms were slightly improved. The significant effect of Madopar was achieved within 1 h after its administration. During the three-month follow-up period, the patient's walking was unsteady. However, the bradykinesia did not progress, and dyskinesia did not recur. The wearing-off was prolonged from 1 h to approximately 2 h, and the overall mental state was better than before the treatment.

## Discussion

As mentioned earlier, the patient, whose case has been described in this paper, was a young male. The disease course lasted for more than 2 years, with an onset of unstable walking and bradykinesia, and gradually increasing resting tremor. The symptoms progressed rapidly. Brain MRI showed mild atrophic changes in the whole brain. The treatment with Madopar was effective. The genetic test results showed two mutation sites of *PLA2G6*, including c.991G > T and c.1427 + 1G > A. On the basis of the present findings and the existing evidence base, this is the first reported EOPD case with mutations in these two sites. The overall prognosis of this disease is poor and with a very high disability and mortality rate. Thus, early identification and treatment are of critical significance to patient prognosis.

Abnormalities in the *PLA2G6* gene are often manifested in various neurodegenerative diseases (Ji et al., 2019), such as infantile neuroaxonal dystrophy (INAD), atypical infantile neuroaxonal dystrophy (ANAD), dystonia parkinsonism (DP), and autosomal recessive EOPD (Cheng et al., 2019). The mechanism of action may be associated with the D331Y and T572I mutations in the *PLA2G6* domain, leading to the reduction of the formation of dopamine neurons and the manifestation of the neuromotor symptom phenotype (Yeh et al., 2021). Several studies have also suggested that potential enzyme activity, DNA methylation, synergistic genetics, and environmental factors may be involved in *PLA2G6* gene-related neurological diseases (Guo et al., 2018), which, therefore, have diverse phenotypes. Among these diseases, EOPD related to the *PLA2G6* gene is more common in young people. The *PLA2G6* gene has been characterized as a locus for parkinsonism with early onset and associated neurological features, particularly dystonia. Many identified mutations have been detected in *PLA2G6* gene-related EOPD (Table 1). However, no other compound with a heterozygous mutation of the *PLA2G6* gene with c.991G > T and c.1427 + 1G > A has been reported. The c.991G > T site of the *PLA2G6* gene is a missense mutation, which may lead to the loss of function of the expressed protein and various motor symptoms (Cheng et al., 2022). In the ClinVar database, the pathogenicity of this variant is classified as pathogenic, and the corresponding disease is EOPD (Shen et al., 2019). Another variant site, c.1427 + 1G > A, is reported to be related to the INAD phenotype (Wan et al., 2022), but the reports of the EOPD phenotype have been scarce. Previous investigations showed that the clinical symptoms of EOPD patients caused by only c.991G > T mutation were relatively mild with typical PD symptoms, no dystonia, and relatively slow disease progression (more than 10 years). In addition, most MRI showed no abnormal signs of cerebral iron deposition (Lu et al., 2012; Xie et al., 2015).

**Table 1:** Summary of all variant sites of EOPD caused by PLA2G6 mutation.

**Nucleotide change**		**Amino acid change**		**Phenotype**	**References**
**Allele 1**	**Allele 2**	**Transcript 1**	**Transcript 2**		
c.1077G > A	c.1670C > T	p.M358IfsX	p.S557L	EOPD	Ching-Chi et al. (2019)
c.991G > T	c.1117G > A	p.D331Y	p.G373R	EOPD	Chen et al. (2018)
c.991G > T	c.1915delG	p.D331Y	p.A639Qfs*27	EOPD	Cheng et al. (2022)
c.967G > A	c.1450G > T	p.V323M	p.D484Y	EOPD	Cheng et al. (2022)
c.991G > T	c.1631T > C	p.D331Y	p.M544T	EOPD	Cheng et al. (2022)
c.991G > T	c.1472 + 1G > A	p.D331Y	–	EOPD	Shen et al. (2019)
c.668C > T	c.1915G > A	p.P223L	p.A639T	EOPD	Tan et al. (2020)
c.991G > T	c.1982C > T	p.D331Y	p.T661M	EOPD	Chen et al. (2018)
c.4C > A	Del Ex3	p.Gln2Lys	p.Leu71_Ser142del	EOPD	Bower et al. (2011)
c.109C > T	c.1078-3C > A	p.Arg37X	–	EOPD	Paisán-Ruiz et al. (2012)
c.109C > T	c.2321G > T	p.Arg37X	p.Ser774Iso	EOPD	Wirth et al. (2017)
c.216C > A	c.1904G > A	p.Phe72Leu	p.Arg635Gln	EOPD	Yoshino et al. (2010)
c.610-1G > T	c.1627C > T	–	p.Arg543Cys	EOPD	Klein et al. (2016)
c.758G > T	c.2341G > A	p.Gly253Val	p.Ala781Thr	EOPD	Wirth et al. (2017)
c.991G > T		p.Asp331Tyr		EOPD	Shi et al. (2011)
c.991G > T	c.1077G > A	p.Asp331Tyr	p.Met358lefsX	EOPD	Lu et al. (2012)
c.1039G > A	c.1670C > T	p.Gly347Arg	p.Ser557Leu	EOPD	Kim et al. (2015)
c.1354C > T	c.1904G > A	p.Gln452X	p.Arg635Gln	EOPD	Yoshino et al. (2010)
c.1495G > A		p.Ala499Thr		EOPD	Yamashita et al. (2017)
c.1547C > T		p.Ala516Val		EOPD	Malaguti et al. (2015)
c.1715C > T		p.Thr572le		EOPD	Paisán-Ruiz et al. (2012)
c.1791delC		p.His597fx69		EOPD	Gui et al. (2013)
c.1894C > T		p.Arg632Trp		EOPD	Sina et al. (2009)
c.1966C > G		p.Leu656Val		EOPD	Gui et al. (2013)
c.2077C > G		p.Leu693Val		EOPD	Gui et al. (2013)
c.2215G > C		p.Asp739His		EOPD	Kamel et al. (2019)
c.2222G > A		p.Arg741Gln		EOPD	Paisan-Ruiz et al. (2009)
c.2239C > T		p.Arg747Trp		EOPD	Paisan-Ruiz et al. (2009)
c.2339A > G		p.Asn780Ser		EOPD	Kauther et al. (2011)
c.2341G > A		p.Ala781Thr		EOPD	Kauther et al. (2011)
c.1634A > G		p.K545R		EOPD	Hua et al. (2022)
c.1495G > A	c.28dupA	p.Ala499Thr	p.Thr10fs	EOPD	Chen et al. (2022)
c.1321T > C	c.856delT	p.C441R	p.S286Pfs12	EOPD	Gao et al. (2020)
c.856delT		p.S286Pfs12		EOPD	Gao et al. (2020)

The c.991G > T of a *PLA2G6* mutant is associated with approximately 30% of the residual protein activity (Shi et al., 2011), which may explain why EOPD patients with only c.991G > T mutation have generally mild disease symptoms and relatively slow disease progression. Although c.991G > T mutation was also present in this case, the condition of the patient was severe, with rapid disease progression. Two years later, the disease had developed into stage 5 according to the Hoehn-Yahr Scale (off-stage), and the case needed special care with his diet and daily life activities. Thus, the disease seriously affected the patients' quality of life, which is inconsistent with findings concerning patients with a single c.991G > T mutation. The patient also differed from other EOPD patients with *PLA2G6* compound heterozygous mutations in terms of cognitive decline and response to madopar. Unlike most patients with *PLA2G6* gene-related EOPD, which is associated with cognitive dysfunction, the patient did not experience cognitive decline. In addition, although the patient began to use madopar at the early stage of the disease, the disorder was not well controlled and progressed rapidly and gradually worsened. During the 2 years of disease course, the bradykinesia symptoms gradually progressed along with the appearance of freezing symptoms, such as inability to move and turn over voluntarily. Most patients have a good response to madopar 5–10 years before onset, which was in contrast to the response of this patient, whose side effects appeared early. The reported here results suggests that these characteristics are associated with the presence of a compound heterozygous mutation of the *PLA2G6* gene with c.991G > T and c.1427 + 1G > A. Several studies have indicated that the rapid progression of the disease in EOPD patients may be related to the fluctuation of the hormone levels in the body (Magrinelli et al., 2022). However, this case had no obvious history of hormone fluctuations. Thus, it was considered that the severe disease with rapid progression was related to the combination of a c.991G > T mutation and a c.1427 + 1G > A mutation. The identification and research of this case laid the foundation for further understanding of *PLA2G6* gene-related neurodegenerative diseases.

## Data availability statement

The raw data supporting the conclusions of this article will be made available by the authors, without undue reservation.

## Ethics statement

Ethical review and approval was not required for the study on human participants in accordance with the local legislation and institutional requirements. The patients/participants provided their written informed consent to participate in this study. Written informed consent was obtained from the individual(s) for the publication of any potentially identifiable images or data included in this article.

## Author contributions

LG, JG, SL, and YL carried out the studies and participated in collecting data. LG and CW performed the statistical analysis and participated in its design. CS, QL, YW, MW, and LH participated in acquisition and draft the manuscript. All authors read and approved the final manuscript.
